# Supplementary material for: Neutralizing Ability of a Single Domain VNAR Antibody: In Vitro Neutralization of SARS-CoV-2 Variants of Concern
Source: Int J Mol Sci. 2022 Oct 14;23(20):12267. doi: 10.3390/ijms232012267 (PMC9603574; doi:10.3390/ijms232012267)
Supplement: Supplementary file 1 [file ijms-23-12267-s001.zip › ijms-1879819-supplementary.pdf]

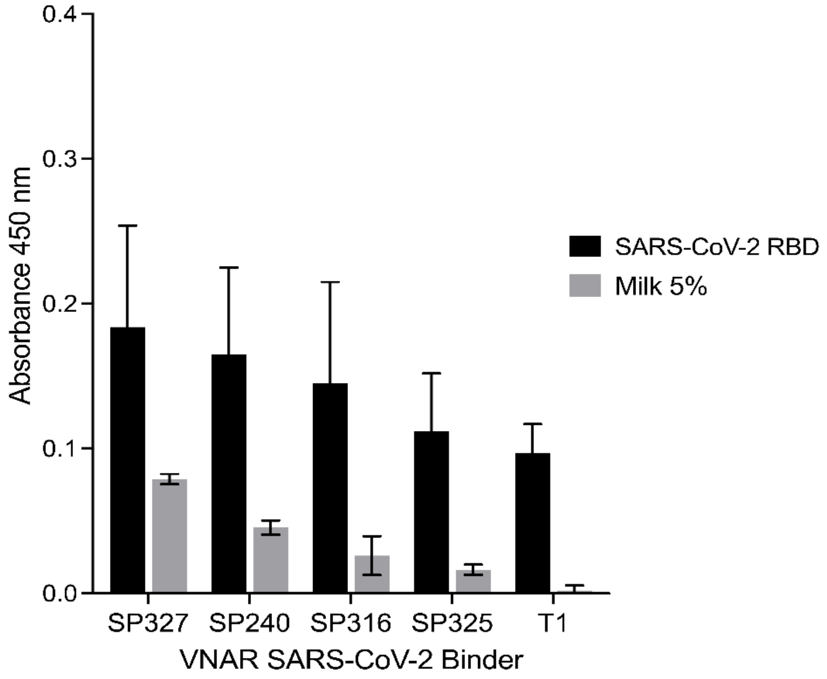

Figure S1. Screening of VNAR SARS-CoV-2 binders by phage ELISA. After the phage display, the isolated clones T1, SP327, SP240, SP316, and SP325 were screened against the RBD of the Spike protein of SARS-CoV-2 by phage ELISA to assess their recognition specificity. Skim milk (5%) was used as a negative control. The binding of each candidate to the RBD was interpreted in absorbance values at 450 nm. The clones that showed an absorbance value at least 1.5 times higher than the negative control (5% milk) were considered positive SARS-CoV-2 binders. The clones SP327, SP240, and SP316 were considered the best SARS-CoV-2 binders. RBD: receptor binding domain of SARS-CoV-2 spike.

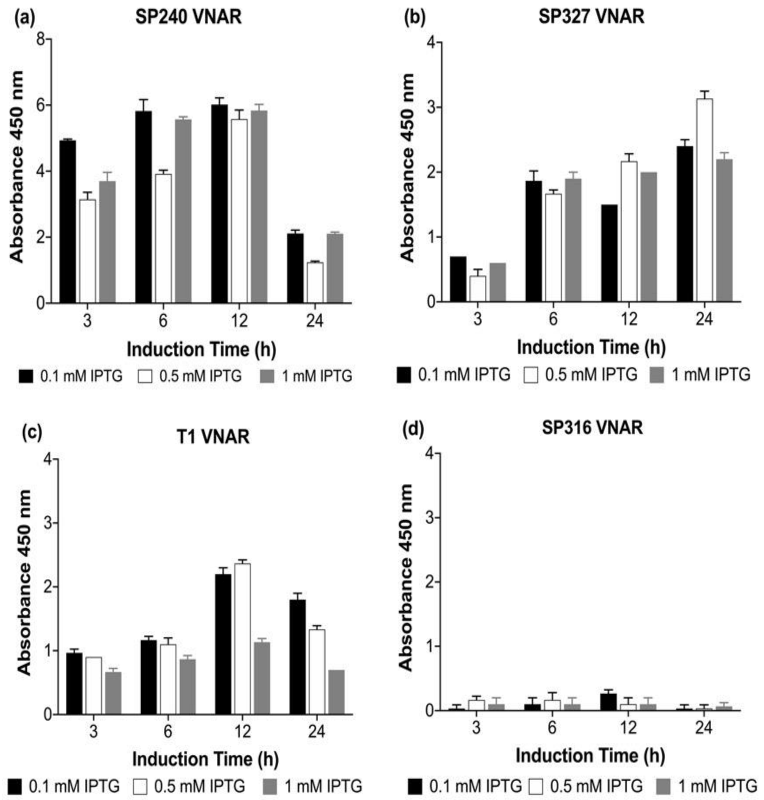

Figure S2. Evaluation of recombinant Variable New Antigen Receptor (VNAR) expression at a small scale. The VNAR proteins were retrieved from the periplasmic extract of *E. coli* BL21 (DE3). The effect of the inductor molecule IPTG in the VNAR proteins expression was evaluated. IPTG was added at concentrations of 0.1, 0.5, and 1 mM, over a total induction period of 24 h. The amount of expressed protein was interpreted in terms of absorbance at 450 nm. SP240 showed the highest expression levels (a), followed by SP327 (b) and T1 (c). SP316 (d) was not successfully retrieved from the periplasmic space.

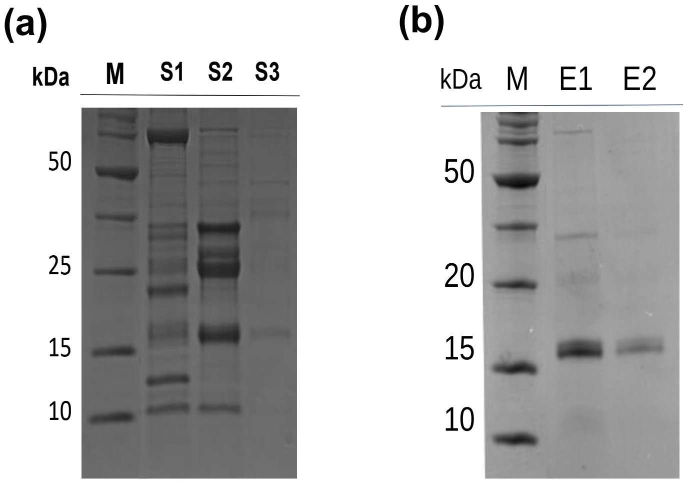

Figure S3. Coomassie-stained sodium dodecyl-sulfate polyacrylamide gel electrophoresis (SDS-PAGE) of the Variable New Antigen Receptor (VNAR) SP240 after Immobilized Metal Affinity Chromatography (IMAC) and immunoaffinity purification. (**a**) Polyacrylamide gel stained with Coomassie blue displayed the eluted proteins after an imidazole gradient of 100 mM (S1), 200 mM (S2), and 300 mM (S3). The positive fractions from IMAC were subjected to immunoaffinity chromatography (**b**). After IMAC, the SP240 protein was revealed by Coomassie staining at the expected size of 15.62 kDa with a few high molecular weight impurities. The thick bands at 15 kDa in wells E1 and E2 correspond to the eluted SP240 protein. Molecular weight marker (M): Broad Range Molecular Protein Marker (Promega).

Table S1. ***In vitro*** **assays for neutralizing activity of VNAR SP240.** Statistical analysis of data obtained after lectures at 450 nm.

	Neutralization potency of VNAR SP240 in A549-ACE2-TMPRSS2								Neutralization potency of VNAR SP240 in Vero E6							
	Delta (B.1.617.2)	± SD	%	N	Omicron (B.1.1.529)	± SD	%	N	Delta (B.1.617.2)	± SD	%	N	Omicron (B.1.1.529)	± SD	%	N
10 µg/ml	1.071	0.006	100	3	1.155	0.001	100	3	0.954	0.028	95	2	1.041	0.001	100	2
5 µg/ml	1.062	0.006	100	3	1.110	0.002	100	3	0.130	0.096	13	2	0.700	0.008	70	2
2.5 µg/ml	0.971	0.005	97	3	1.055	0.032	100	3	-0.050	0.005	0	2	-0.009	0.047	0	2
1.25 µg/ml	0.685	0.015	69	3	0.941	0.062	94	3	-0.040	0.025	0	2	0.016	0.0005	0	2
0.625 µg/ml	0.415	0.023	42	3	0.821	0.027	82	3	-0.020	0.006	0	2	-0.128	0.017	0	2
0.312 µg/ml	0.312	0.013	31	3	0.527	0.029	53	3	-0.007	0.012	0	2	-0.201	0.001	0	2
0.156 µg/ml	0.178	0.034	18	3	0.483	0.049	48	3	0.002	0.008	0	2	-0.093	0.021	0	2
0.078 µg/ml	0.095	0.067	10	3	0.169	0.024	17	3	-0.040	0.016	0	2	-0.155	0.027	0	2

Table S2. ***In vitro*** **assays for neutralizing activity of VNAR P98Y.** Statistical analysis of data obtained after lectures at 450 nm.

	Neutralization potency of VNAR P98Y in A549-hACE2-TMPRSS2											
	SARS-CoV-2 D614G	± SD	%	N	Delta (B.1.617.2)	± SD	%	N	Omicron (B.1.1.529)	± SD	%	N
10 µg/ml	0.217	1.195	21.7	2	0.471	1.600	47.1	2	0.489	0.405	48.9	2
5 µg/ml	0.103	0.825	10.3	2	0.174	0.580	17.4	2	0.286	0.415	28.6	2
2.5 µg/ml	0.032	0.015	3.2	2	-0.025	0.165	0.0	2	0.067	0.250	6.7	2
1.25 µg/ml	-0.003	0.125	0.0	2	0.008	0.040	0.0	2	-0.025	0.650	0.0	2
0.625 µg/ml	0.009	0.390	0.0	2	-0.006	0.240	0.0	2	-0.014	0.275	0.0	2
0.312 µg/ml	-0.040	0.100	0.0	2	-0.036	0.325	0.0	2	-0.013	0.230	0.0	2
0.156 µg/ml	-0.029	0.215	0.0	2	-0.035	0.755	0.0	2	-0.005	0.390	0.0	2
0.078 µg/ml	0.008	0.350	0.0	2	0.003	0.050	0.0	2	-0.014	0.070	0.0	2

	Neutralization potency of VNAR P98Y in Vero E6											
	SARS-CoV-2 D614G	± SD	%	N	Delta (B.1.617.2)	± SD	%	N	Omicron (B.1.1.529)	± SD	%	N
10 µg/ml	0.492	0.045	49.2	2	0.593	0.038	59.3	2	0.553	0.044	55.3	2
5 µg/ml	0.288	0.005	28.8	2	0.413	0.033	41.3	2	0.279	0.003	27.9	2
2.5 µg/ml	0.282	0.004	28.2	2	0.419	0.046	41.9	2	0.202	0.001	20.2	2
1.25 µg/ml	0.214	0.024	21.4	2	0.281	0.011	28.1	2	0.218	0.028	21.8	2
0.625 µg/ml	0.175	0.007	17.5	2	0.256	0.088	25.6	2	0.130	0.023	13.0	2
0.312 µg/ml	0.064	0.020	6.4	2	0.170	0.002	17.0	2	0.109	0.010	10.9	2
0.156 µg/ml	0.024	0.008	2.4	2	0.023	0.006	2.3	2	0.054	0.008	5.4	2
0.078 µg/ml	-0.015	0.009	0.0	2	-0.005	0.003	0.0	2	-0.026	0.009	0.0	2
